# Readmission and overstay after day case nasal surgery

**DOI:** 10.1186/1472-6815-4-2

**Published:** 2004-10-22

**Authors:** Gurminder Singh, David McCormack, David R Roberts

**Affiliations:** 1 Guy's, King's, & St. Thomas' School of Medicine, London, UK; 2 3 Bakewell Close, Mickleover, Derby DE3 9JS UK; 3 The ENT & Audiology Department, 3^rd ^Floor Thomas Guy House, Guy's Hospital, SE1 9RT UK

## Abstract

**Background:**

A readmission is classified as a patient necessitating readmission to hospital due to a post-operative complication following discharge. An overstay however, is classified as a patient having to stay longer than the planned duration in hospital (not having been discharged in the interim) due to a post-operative complication. This study aims to investigate patient-related factors that predispose to readmission or overstay and thus make recommendations to decrease the likelihood of readmission or overstay.

**Method:**

In this retrospective study 312 'day-case nasal procedures', were selected from a total cohort of 4274 ENT patients over a 17-month period. This sub-group was investigated for a range of demographic factors including, age, gender and ethnicity with regards to their relationship to readmission rates and overstay frequency and duration.

**Results:**

The rates were 2.88% and 9.62% for readmission and overstay respectively. The total number of days spent in hospital as a result of readmission was 27. Epistaxis was the leading cause for readmission/overstay (28.9%) followed by high levels of post-operative pain preventing them from being discharged (23.7%). All procedures in this study had readmission rates that were below those recommended in the guidelines set by the Royal College of Surgeons of England. Women overstayed significantly longer (t = 1.65, p < 0.05) than men.

**Conclusions:**

Suitable candidates for day-case ENT surgery highlighted by this study include healthy individuals between the ages of 20 and 60. Operating in the morning would increase the immediate post-operative recovery time, which may reduce the numbers of patients who complain of high levels of pain at the time of discharge. Procedures such as septorhinoplasty being performed routinely in the ambulatory setting require additional research into more effective methods of pain control. Standards need to be improved so that the causes of overstay and readmission are clearly identifiable in patient records.

## Background

Day-case surgery has rapidly expanded as a cost-effective and resource-conserving surgical intervention to the point that well in excess of two million operations are performed in a day-case/ambulatory setting in the United Kingdom alone each year [1]. Day-case surgery is based on operating on a patient and aiming to discharge them on the same day. Ear, nose and throat procedures account for a significant proportion of these ambulatory procedures and include operations such as functional endoscopic sinus surgery and rhinoplasty. It has been suggested that day-case surgery should be confined to those procedures where less than 3% of patients require admission post-operatively [1]. Septorhinoplasty is generally considered to be a traumatic procedure with risks of epistaxis and periorbital haematoma. However, the decision to perform septorhinoplasty in a day case setting may be made on the basis of its cost-effectiveness and rapid post-operative recovery in suitable operative candidates. Through the NHS Plan, the Department of Health has stated its aim of three-quarters of operations will being performed on a day case basis within the next decade [2]. It states that in these procedures there will be no overnight stay required so that traditional waiting lists for surgery will 'become a thing of the past'.

A King's College Hospital Study commissioned by the Department of Health has suggested that day-case surgery may not be saving the NHS money [3]. However, previous reports have advocated day case septal surgery as a safe and effective practice [4-6]. Furthermore, a subjective patient based study showed that septoplasty is generally acceptable to the patient in terms of pain and overall satisfaction parameters [5,7].

Studies have shown that day-case septorhinoplasty is associated with a low complication rate and is a safe and acceptable procedure provided that stringent patient selection criteria are adhered to [8]. A specific area of dissatisfaction previously identified is inadequate pain control following discharge and this may lead to higher costs for the general practitioner. However, a recent study investigating parental satisfaction with 100 paediatric otorhinolaryngology day-case procedures concluded that with careful patient selection the degree of satisfaction with day surgery is high for a wide variety of procedures [9,10].

There have been no studies performed, which have directly investigated the 'patient demographic factors' which predict the likelihood readmission or overstaying the electively planned period in hospital following day-case ENT operations. This is of importance as it would be beneficial to know if certain groups of patients require more rigorous screening pre-operatively in an attempt to reduce readmission and 'overstay' rates. By identifying this cohort of patients, the NHS could save substantial amounts of acute and primary care expenditure in the backdrop of ever-rising day-case ENT procedure numbers. Many studies have suggested that ENT procedures currently performed as overnight cases could be performed as day-cases provided strict criteria are applied in the selection of patients [1,11]. However, none of these studies provide a clear description as to which patient groups are potentially unsuitable for undergoing day-case procedures based upon their predisposition to require readmission or stay in hospital longer than electively planned.

Previous data fails to distinguish between readmissions and overstays following nasal day-case surgery [11]. They also fail to investigate demographic indices such as age, gender, and ethnicity [5]. The present study has been conducted in the climate of an ever-advancing quality-driven clinical environment in which meticulous patient selection is vital in optimal patient post-operative care and recovery. This study of 312 elective day-case nasal operations selected from a cohort of 4274 ENT patients investigated a range of patient variables that influenced readmission and overstay frequency and duration.

This study investigates departmental day-case nasal surgery at Guy's hospital and aims to determine:

a) If it has readmission rates below that of the accepted standard for day-case surgery stipulated by the Royal College of Surgeons of England.

b) If readmission rate and unplanned overnight stay are related to epidemiological characteristics of the sample such as age, gender, and ethnicity.

and to report results in the context of clinical management by making recommendations to improve the readmission rates in each category proposed.

## Method

This is a retrospective study in which the total number of ENT operations performed between the period of 3^rd ^January 2002 and 28^th ^June 2003 were investigated. All ENT patients operated on during this period were selected from the Guy's & St. Thomas' NHS Trust database. The hospital database has a limited range of broad categories and this can limit interpretation of results thus all available patient notes were also reviewed.

All planned day-case procedures were selected from the total cohort of elective ENT operative procedures. The day-case procedures were then further narrowed down to 'nasal day-case procedures'.

Firstly the total number of readmission episodes, overstay frequency and duration in the 'day-case nasal procedures' were calculated. A readmission is classified as a patient necessitating readmission to hospital due to a post-operative complication following discharge. An overstay however, is classified as a patient having to stay longer than the planned duration in hospital (not having been discharged in the interim) due to a post-operative complication. The groups of nasal operations of interest in this study in the light of previous studies are (septo)rhinoplasty, excision of lesion, polypectomy, sinus operation and other (which includes procedures such as intranasal ethmoidectomy, and division of adhesions of turbinate of nose etc).

A range of demographic factors including, age, gender and ethnicity were investigated with regards to their relationship to readmission rates and overstay frequency and duration. Statistical analysis including Chi-square tests, t-tests and ANOVA were performed on the above variables.

## Results

The total number of ENT operations performed between the period of 3^rd ^January 2002 and 28^th ^June 2003 were 4274. From this cohort 501 operations (11.72%) were planned as elective ENT day-case procedures. This selected sub-sample of 501 day-cases was further narrowed down to those, which pertained to 'nasal procedures' and this group numbered 312 (62.28%). There were a total of 9 readmission episodes following 'day-case nasal procedures' during the 17-month period, which equates to a readmission rate of 2.88%. The total number of days spent in hospital as a result of readmission was 27. All patients readmitted following day-case nasal procedures were male (Fischer Exact Test = 4.41, p < 0.05). The nasal operations were grouped into (septo)rhinoplasty, excision of lesion, polypectomy, sinus operation and other.

Those patients that were required to stay longer than electively planned i.e. one day, are termed by the Guy's & St. Thomas' NHS Trust and indeed in this study as 'over-stays'. The minimum duration of overstay in our sample was one day and the maximum was 2 days. The total incidence of patients overstaying was 30 (overstay rate = 9.62%) during the timeframe studied and this equates to a total of 48 overstayed days.

### Cause of readmission and overstay

Epistaxis was the leading cause for readmission/overstay *n *= 11/38 (28.9%) followed by levels of post-operative pain unacceptable to the patient and thus preventing them from being discharged (23.7%).

### Type of procedure

**Figure 1:**
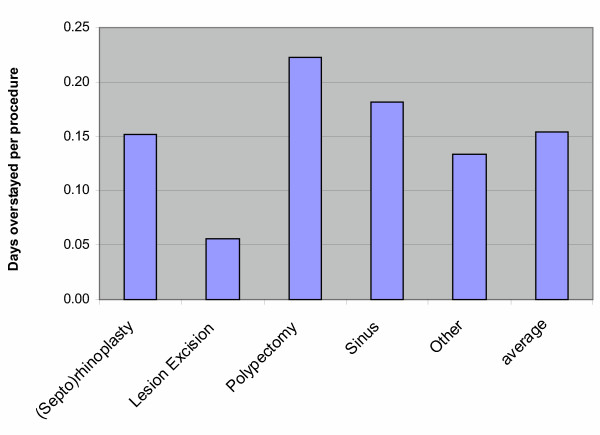
Days overstayed as a fraction of the procedures performed.

The readmission rate for (septo)rhinoplasty is 1.38% and the overstay rate is 9.22%. From the analysis of days overstayed expressed as a fraction of the number of procedures performed, polypectomy and sinus operations have the highest number of days overstayed per procedure. Although the (septo)rhinoplasty overstay rate is 9.22% the number of days overstayed expressed in the context of the number of actual (septo)rhinoplaties performed is equal to the average (see Figure 1).

### Gender

Women overstayed significantly longer (t = 1.65, p < 0.05) than men.

### Age

The frequency of readmission is highest in those patients aged <20 followed by those in the 60–70 year age category. Although the patients aged above 70 years have the lowest readmission frequency, the sample size is low (n = 5) and thus the 30–40 year age category is more reliable a sample representative of a low readmission frequency. Analysis of the age of patients and frequency of day-case procedures revealed that the group containing those patients aged between 30–40 underwent the greatest number of nasal day-case procedures. However, when considering the number of overstays in terms of the numbers of procedures performed, the 30–40 age category has the lowest overstay duration as opposed to the >70 which has the highest.

### Ethnicity

Analysis of variance (ANOVA) shows that the difference in overstay rates between ethnic groups did not reach statistical significance (F = 1.22, p = 0.40).

## Discussion

In July 2000, the Department of Health published that 75% of surgical procedures carried out by the NHS should be performed in a day-case setting by 2010. This initiative was supported by the both The Royal College of Surgeons of England and The Royal College of Physicians of England.

Within the period of the current study there were a total of 4274 ENT operations performed at Guy's during the time-frame of investigation. Of this group 501 (11.72%) were planned as elective ambulatory procedures with the intention of same day discharge. Primarily the government target was ambiguous with regard to the types of procedure for which it aimed to conduct on a routine day-case basis. Whilst it is conceivable that some ENT procedures such as myringotomy could be performed safely as a day-case procedure, more complex and involved procedures such as deep exploration of the neck and tumour excision would be unsuitable for such rapid discharge. With no clear guidelines as to which procedures are suitable for the day-case setting, the responsibility of advancing the boundaries of ambulatory surgery rests on the clinicians, with the patients' wellbeing being at the forefront of interest.

The readmission in this study is 2.88%. These readmission rates are lower than those reported in the contemporary literature [4,8,11]. The readmission rate for (septo)rhinoplasty is 1.38% and the overstay rate is 9.22%. These rates are lower than those stipulated by the Royal College of Surgeons of England which pertain to day-case surgery in general (2–3%) as opposed to ENT cases per se.

Epistaxis was the leading cause for readmission/overstay (28.9%) followed by levels of post-operative pain unacceptable to the patient and thus preventing them from being discharged (23.7%). The cause of epistaxis was not recorded in the notes nor was the reason behind why some patients experience more pain than others. These results are in contrast to previous subjective patient based studies, which show that septorhinoplasty is generally acceptable to the patient in terms of pain and overall satisfaction parameters [5]. Women overstayed significantly longer (t = 1.65, p < 0.05) than men. A similar phenomenon has been reported in a study of cardiothoracic patient readmissions [12]. In this study the female predilection to increased in-hospital recovery time was attributed to various gender-associated factors.

Age is not a contraindication for ENT day-case surgery. The low readmission rate of patients over 70 years of age could be attributed to a number of factors including, heightened caution of the clinician in patient discharge, a low sample number and age-related variation of procedure type are dominant.

There have been no previous studies, which have investigated the effects of ethnicity on readmission rates or overstay duration. The current study shows that mixed race patients followed by black patients have the highest number of days overstayed per procedure. Analysis of variance (ANOVA) shows that there is no significant difference between ethnic groups and the length of overstay (F = 1.22, p = 0.40).

## Conclusions

Day-case procedures should be performed on suitable candidates on a sound clinical basis. This includes meticulous patient selection, both on the part of the surgeon and the health care professionals in the preoperative assessment. Suitable candidates for day-case ENT surgery highlighted by this study include healthy individuals between the ages of 20 and 60. A protocol for day-case surgery does exist and this may need revision.

In the clinical setting however, epistaxis needs to be made a clinical priority by ensuring that all levels of healthcare professionals are aware of its causes and its effective immediate management. We suggest from experience that operating in the morning would increase the immediate post-operative recovery time, which may reduce the numbers of patients who complain of high levels of pain at the time of discharge. Procedures such as septorhinoplasty being performed routinely in the ambulatory setting require additional research into more effective methods of pain control. Clinical administration standards need to be improved so that the causes of overstay and readmission are clearly identifiable in patient records.

## Competing interests

The authors declare that they have no competing interests.

## Authors' contributions

Mr. Gurminder Singh (first and corresponding author) performed the data collection and wrote the paper with assistance from Mr. David McCormack (second author). Mr. David Roberts supervised the study and reviewed the completed manuscript.

## Pre-publication history

The pre-publication history for this paper can be accessed here:


